# Molecular crosstalk between ferroptosis and apoptosis: emerging role of ER stress-induced p53-independent PUMA expression

**DOI:** 10.18632/oncotarget.23046

**Published:** 2017-12-08

**Authors:** Se Hoon Hong, Dae-Hee Lee, Young-Sun Lee, Min Jee Jo, Yoon A Jeong, William T. Kwon, Haroon A. Choudry, David L. Bartlett, Yong J. Lee

**Affiliations:** ^1^ Department of Surgery, School of Medicine, University of Pittsburgh, Pittsburgh, PA 15213, USA; ^2^ Brain Korea 21 Program for Biomedicine Science, Korea University College of Medicine, Korea University, Seoul 02841, Republic of Korea; ^3^ Division of Oncology/Hematology, Department of Internal Medicine, College of Medicine, Korea University Medical Center, Korea University, Seoul 08308, Republic of Korea

**Keywords:** ferroptosis, apoptosis, PUMA, ER, p53

## Abstract

Ferroptosis is a type of programmed cell death that depends on iron and is characterized by the accumulation of lipid peroxides. In the present study, we investigated the nature of the interplay between ferroptosis and other forms of cell death such as apoptosis. Human pancreatic cancer PANC-1 and BxPC-3 and human colorectal cancer HCT116 cells were treated with ferroptotic agents such as erastin and artesunate (ART) in combination with the apoptotic agent tumor necrosis factor-related apoptosis-inducing ligand (TRAIL). We observed synergistic interaction of erastin or ART with TRAIL as determined by cell death assay, caspase activation, poly [ADP-ribose] polymerase 1 (PARP-1) cleavage, flow cytometry analysis, and lipid peroxidation assay. Moreover, erastin and ART induced endoplasmic reticulum (ER) stress and promoted p53 upregulated modulator of apoptosis (PUMA) expression via C/EBP-homologous protein (CHOP). Synergy of erastin/ART and TRAIL was abolished in PUMA-deficient HCT116 cells and CHOP-deficient mouse embryonic fibroblasts, but not in p53-deficient HCT116 cells. The results suggest the involvement of the p53-independent CHOP/PUMA axis in response to ferroptosis inducers, which may play a key role in ferroptotic agent-mediated sensitization to TRAIL-induced apoptosis.

## INTRODUCTION

Several processes such as ferroptosis, apoptosis, necrosis, and autophagy are the primary mechanisms of cell death. Each type of biological death leaves a biochemical and morphological fingerprint [1]. However, emerging evidence suggests that these different types of cell death often share common pathways [2].

Ferroptosis is a unique iron-dependent form of regulated cell death [3]. The term was coined in 2012 by studies identifying several genes responsible for ferroptosis, including those associated with amino acids such as glutamate and cysteine, and lipid metabolism [4–6]. Ferroptosis triggered by these genes can be inhibited by chemical compounds such as the cystine/glutamate antiporter (System X_C_^−^) inhibitor erastin, the glutathione (GSH) synthesis inhibitor buthionine sulfoximine (BSO), the glutathione-dependent antioxidant enzyme glutathione peroxidase 4 (GPX4) inhibitor (1S, 3R)-RSL3, and the glutathione S-transferase inhibitor artesunate (ART) [3, 7–11]. Iron-dependent accumulation of lipid reactive oxygen species (ROS) and depletion of plasma membrane polyunsaturated fatty acids have been well known to result in this lethal event [3, 4, 12, 13]. Differences in genetic makeup among cancer cells affect the pharmacodynamic response of ferroptotic agents. High level RAS-RAF-MEK pathway activity or p53 expression may elevate generation of ROS through mitochondrial voltage-dependent anion channel 2/3 (VDAC2/3) or inhibit cystine uptake, respectively, and sensitize cancer cells to ferroptosis [14–19]. Conversely, iron chelators (e.g., deferoxamine) and lipid peroxidation inhibitors (e.g., liproxstatin, ferrostatin, and zileuton) are known to suppress ferroptosis and block pathological cell death events in the brain, kidney, and other tissues [10, 20–23].

Since its discovery in 1995, tumor necrosis factor-related apoptosis-inducing ligand (TRAIL) has sparked growing interest among oncologists due to its remarkable ability to induce apoptosis in malignant human cells, but not in most normal cells [24, 25]. TRAIL initiates the extrinsic pathway by binding to death receptors (DRs) such as DR4 and DR5 and induces the apoptotic signal. Activation of death domains (DDs) leads to formation of the death-inducing signaling complex (DISC) [26]. Caspase-8 recruitment and its activation at the DISC leads to further triggering of signaling molecules downstream, which results in the activation of executioner caspase-3, -6, and -7, which culminates in apoptotic death [27]. Activated caspase-8 also cleaves a pro-apoptotic molecule, Bid; truncated Bid (tBid) translocates to the mitochondria and induces Bax (Bcl-2-associated X protein) and Bak (Bcl-2 homologous antagonist killer) oligomerization [28, 29]. Oligomerized Bax and Bak’s insertion into the mitochondrial outer membrane, permeabilization, and depolarization of the mitochondria promote cytochrome *c* release [30]. Released cytochrome *c* facilitates the formation of the Apaf1 (apoptosis signal-regulating kinase)/caspase-9 apoptosome, which activates caspase-9 and subsequently, caspase-3 [31].

TRAIL-induced cytotoxicity can be modulated by various agents such as chemotherapeutic drugs [32–34], ionizing radiation [35], other cytokines [36], and matrix metalloprotease inhibitors [37]. In this study, we observed a synergistic interaction between TRAIL and ferroptotic agents. A combined treatment of ART/erastin with TRAIL markedly enhanced TRAIL-induced apoptosis. Our studies suggest that these synergistic effects are due to endoplasmic reticulum (ER) stress-induced p53-independent PUMA (p53 upregulated modulator of apoptosis) expression.

## RESULTS

### Ferroptosis inducers ART and erastin enhance TRAIL-induced apoptosis

**Figure 1:**
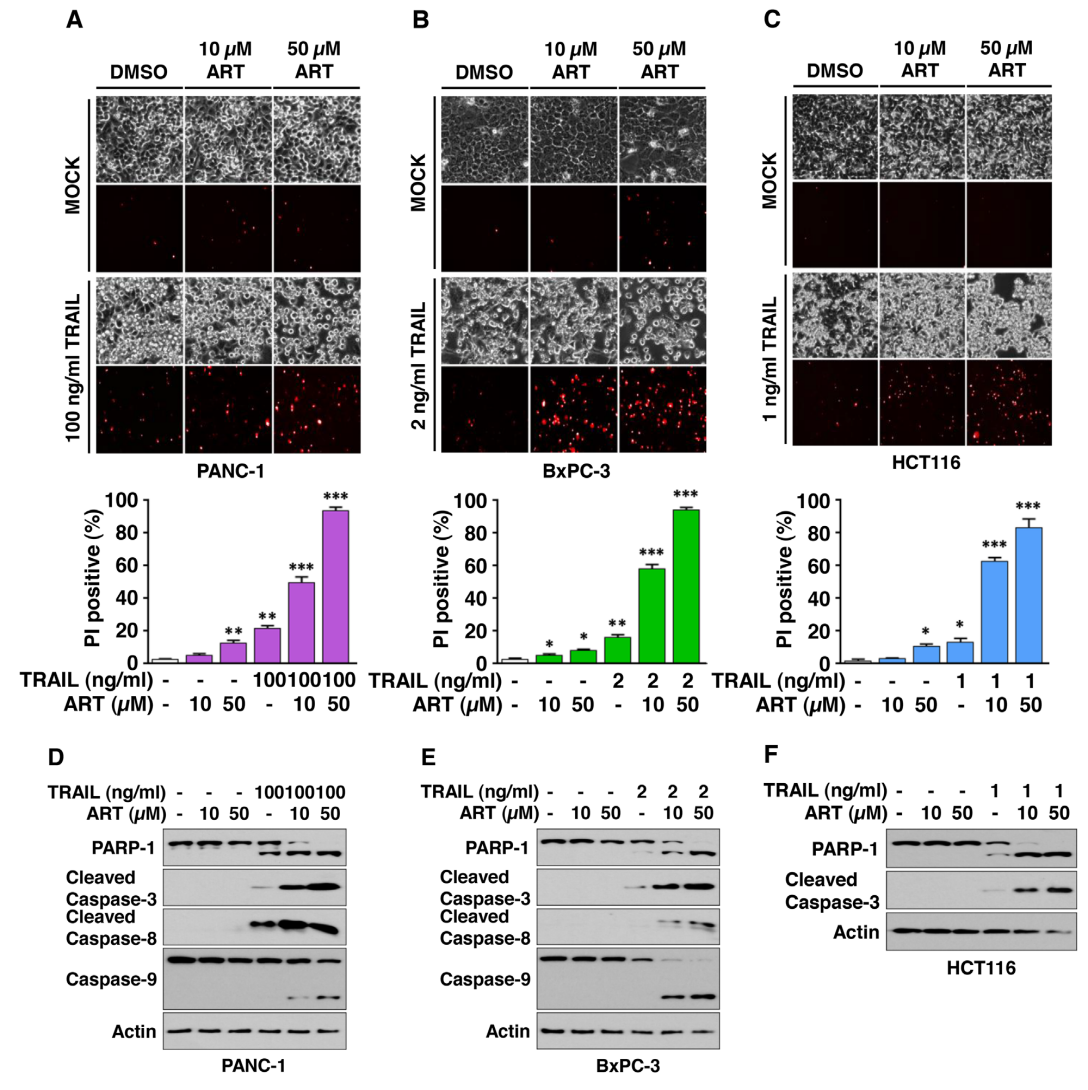
Artesunate (ART) promotes TRAIL-induced apoptosis **(A-F)** PANC-1 (A), BxPC-3 (B), and HCT116 (C) cells were pretreated with ART (10 or 50 μM) for 20 h and then exposed to TRAIL (PANC-1, 100 ng/ml; BxPC-3, 2 ng/ml; HCT116, 1 ng/ml) for an additional 4 h. The cells were stained with propidium iodide (PI). Phase-contrast images or fluorescence images were visualized under a light or fluorescence microscope, respectively (upper panels). Representative images are shown (magnification, X200). Cell death was determined by counting PI-stained cells and plotted (lower panels). Error bars represent the mean ± SD from triplicate experiments. For statistical analysis, Student’s *t*-test (two-sided, paired) was used. *p*-values: ^*^, 0.05; ^**^, 0.01; ^***^, 0.001. Cell lysates of PANC-1 (D), BxPC-3 (E), and HCT116 (F) cells were analyzed with immunoblotting assay using indicated antibodies.

**Figure 2:**
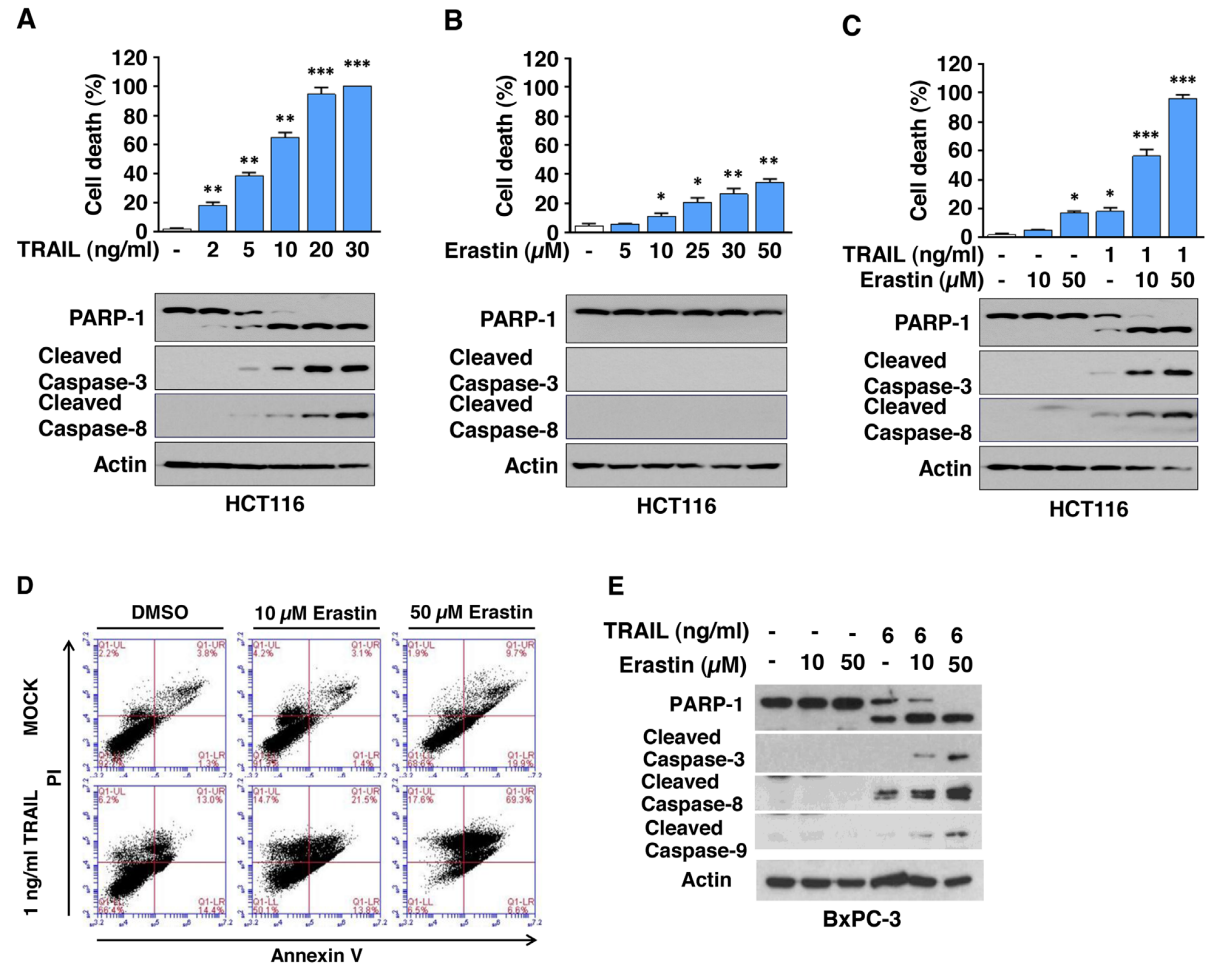
Erastin promotes TRAIL-induced apoptosis **(A** and **B)** HCT116 cells were treated with various doses of erastin for 24 h (A) or TRAIL for 4 h (B) and then the cell death rate was determined, respectively (upper panels). Cell lysates were analyzed with immunoblotting assay using indicated antibodies (lower panels). **(C** and **D)** HCT116 cells were pretreated with erastin (10 or 50 μM) for 20 h and then exposed to TRAIL (1 ng/ml) for an additional 4 h. Cell death was determined by counting and plotted. Whole-cell extracts were then analyzed with immunoblotting assay using indicated antibodies (C). The cells were stained with annexin V and PI and then analyzed using flow cytometry (D). **(E)** BxPC-3 cells were pretreated with erastin (10 or 50 μM) for 20 h and then exposed to TRAIL (6 ng/ml) for an additional 4 h. Whole-cell extracts were then analyzed with immunoblotting assay using indicated antibodies.

To determine whether ferroptotic agents are cytotoxic to human cancer cells, human pancreatic cancer PANC-1 and BxPC-3 and human colon cancer HCT116 cells were treated with various doses of ART. We observed dose-dependent effects of ART on cytotoxicity (Figure 1). Next, we examined the effect of combinatorial treatment (ART + TRAIL) on cytotoxicity. As shown in Figure 1A-1C, a synergistic cytotoxic effect was observed with the combinatorial treatment compared with any single treatment (p<0.001). Confirmation was obtained by combination index (CI) analysis as shown in Table 1; there was strong or moderate synergy of ART in combination with TRAIL, especially in the high dose group in the three cell lines. We further investigated whether the combinatorial treatment-induced cytotoxicity was associated with apoptosis. Data from the immunoblotting assay demonstrate that the synergistic effects were due to an increased activation (cleavage) of caspase 8/9/3 and thus, the hallmark feature of apoptosis, PARP (poly (ADP-ribose) polymerase) cleavage (Figure 1D-1F). Similar results were observed with erastin in combination with TRAIL-treated HCT116 cells. We observed a dose-dependent apoptotic effect of TRAIL and ferroptotic effect of erastin (Figure 2A and 2B). The synergistic cytotoxicity was observed when cells were treated with erastin in combination with TRAIL (Figure 2C and Table 1). Data from the immunoblotting assay and flow cytometry assay demonstrate that the combined treatment-induced cytotoxicity was associated with apoptosis as evident by increased PARP cleavage (Figure 2C) and apoptotic cells at the upper right quadrant of the plots (Figure 2D). An increase in apoptosis was also observed in BxPC-3 cells during treatment with TRAIL and erastin (Figure 2E). These results indicate that synergistic induction of cytotoxicity by combined treatment of erastin or ART with TRAIL is mediated by an increase in apoptosis.

**Table 1:** Combination index (CI) for ART/erastin and TRAIL

Combination therapy			Combination index (CI)
TRAIL (ng/ml)	Erastin (μM)	ART (μM)	HCT116
1	10	-	0.327^**^
1	50	-	0.161^***^
1	-	10	0.301^**^
1	-	50	0.367^**^

^*^: slight synergy; ^**^: moderate synergy; ^***^: strong synergy

### Ferroptotic agent-induced lipid peroxidation is not changed or influenced by the combination treatment of erastin or ART with TRAIL

**Figure 3:**
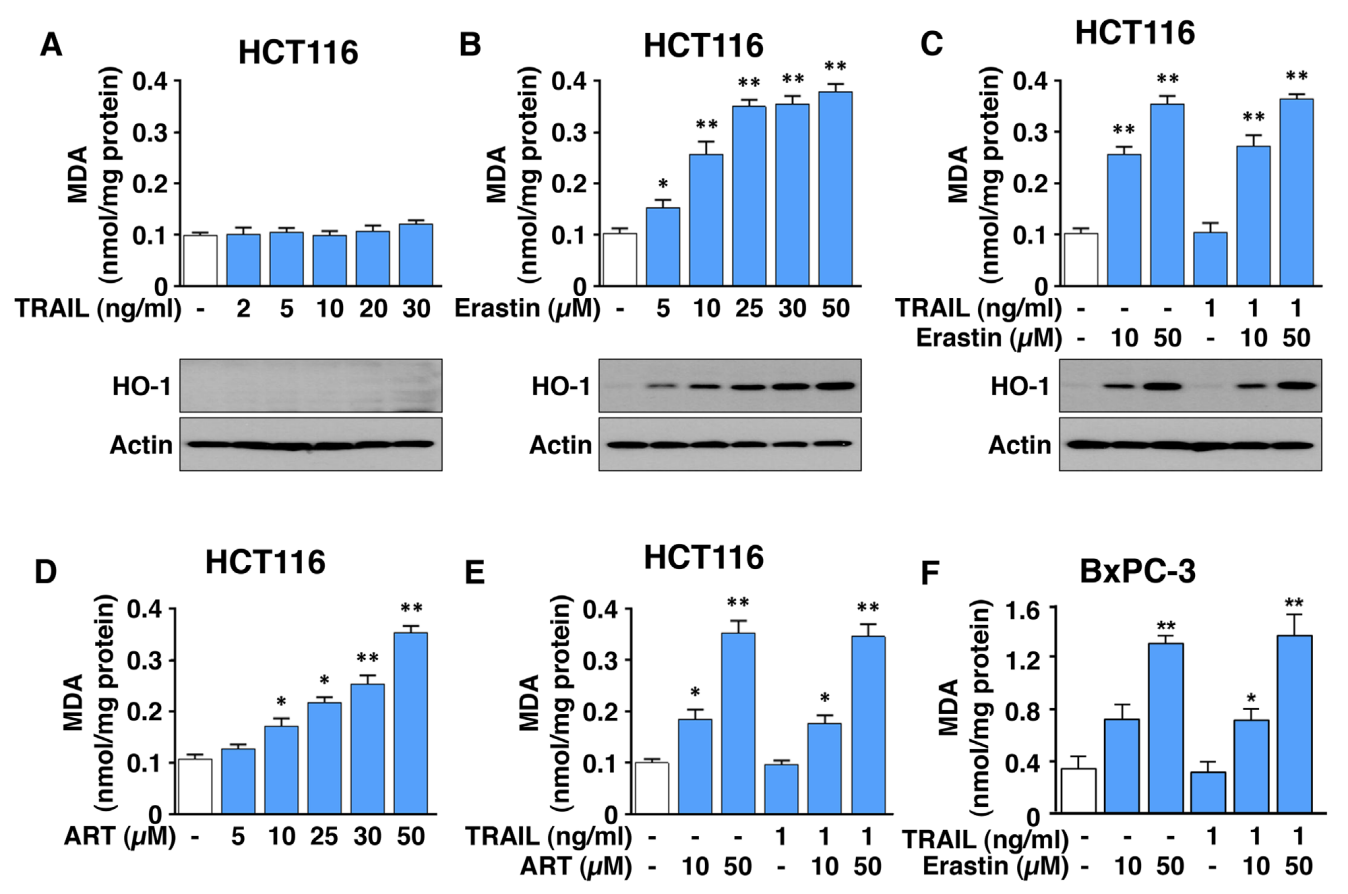
ART and erastin, but not TRAIL, induce lipid peroxidation **(A-C)** HCT116 cells were treated with various doses of erastin for 24 h (A) or TRAIL for 4 h (B). Cells were pretreated with erastin (10 or 50 μM) for 20 h and then exposed to TRAIL (1 ng/ml) for an additional 4 h (C). Lipid peroxidation (upper panels) and heme oxygenase (HO-1, lower panels) levels were analyzed by malondialdehyde (MDA) assay and immunoblotting assay, respectively. **(D** and **E)** HCT116 cells were treated with various doses of ART (D) or pretreated with ART (10 or 50 μM) for 20 h and then exposed to TRAIL (1 ng/ml) for additional 4 h (E). **(F)** BxPC-3 cells were pretreated with erastin (10 or 50 μM) for 20 h and then exposed to TRAIL (1 ng/ml) for additional 4 h. MDA levels were determined and plotted. Error bars represent the mean ± SD from triplicate experiments. For statistical analysis, Student’s *t*-test (two-sided, paired) was used. *p*-values: ^*^, 0.05; ^**^, 0.01.

Since we observed that the combinatorial treatment-induced synergistic cytotoxicity was due to an increase in apoptosis, we examined whether the combinatorial treatment enhances ferroptosis by analysis of malondialdehyde (MDA), a lipid peroxidation marker. As shown in Figure 3A, and 3D, ferroptotic agents, but not TRAIL, induced lipid peroxidation in a dose-dependent manner in HCT116 cells. The combined treatment of TRAIL and erastin/ART did not enhance ferroptotic agent-induced lipid peroxidation (Figure 3C and 3E). Similar results were observed in BxPC-3 cells (Figure 3F). Similar to the lipid peroxidation result, erastin, but not TRAIL, induced heme oxygenase-1 (HO-1) expression, a marker of oxidative stress, in a dose-dependent manner (Figure 3B). The combined treatment of TRAIL and erastin did not enhance ferroptotic agent-induced HO-1 expression.

### Ferroptotic agents induce ER stress

**Figure 4:**
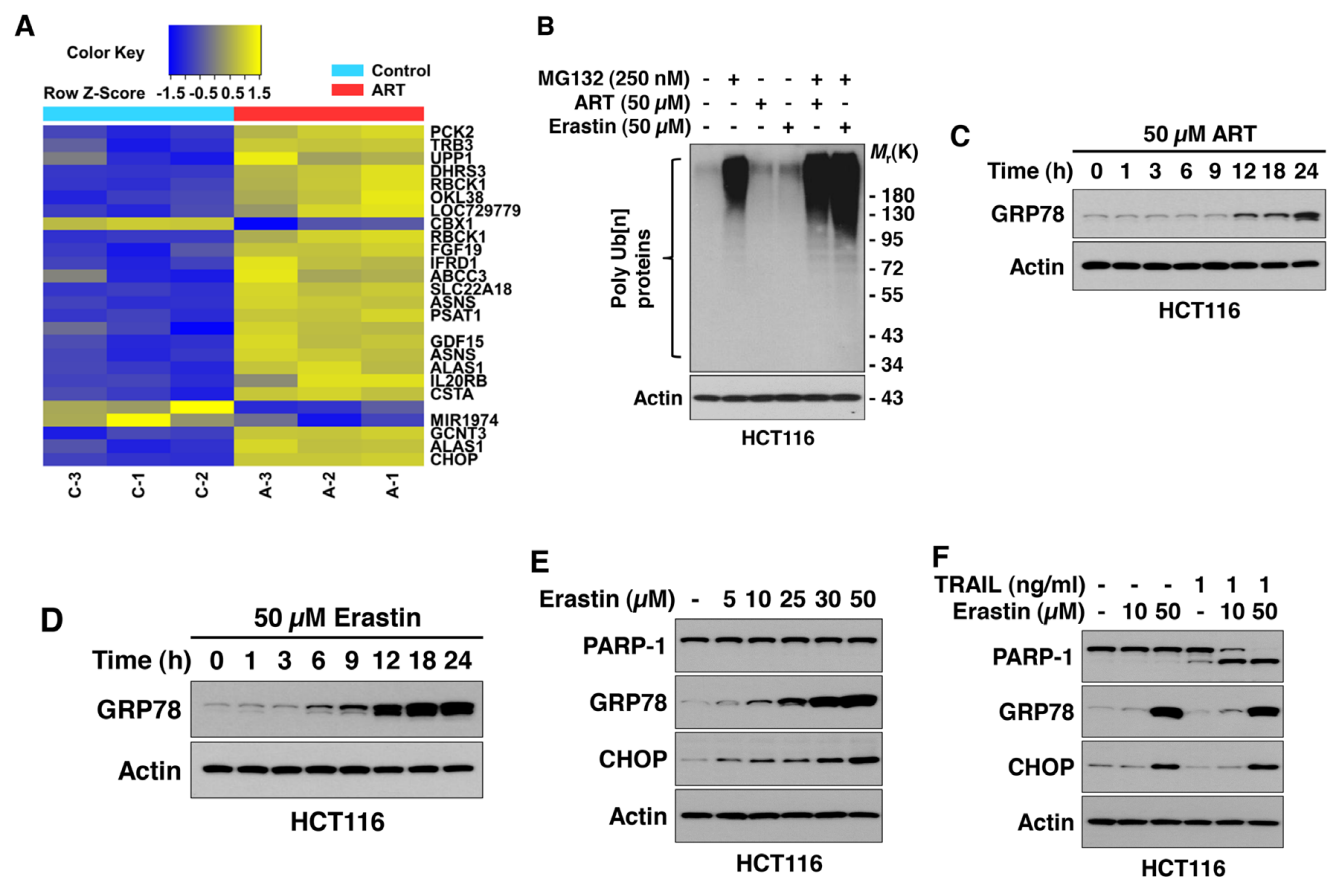
Ferroptotic agents induce ER stress in HCT116 cells **(A)** Microarray assay for detection of ART-induced gene expression. Cells were treated with 50 μM ART for 24 h and triplicate Illumina gene expression microarrays were performed with BeadArray microarray technology. **(B)** Ferroptotic agents induce the unfolded protein response (UPR). Cells were treated with 50 μM ART or 50 μM erastin for 24 h in the presence/absence of 250 nM MG132. Cell lysates were subjected to immunoblotting analysis using FK2 antibody specific to ubiquitin-conjugated proteins. Actin was used to confirm equal amounts of proteins loaded in each lane. **(C** and **D)** Cells were treated with ART (50 μM, B) or erastin (50 μM, C) for various times (1-24 h). Whole-cell extracts were analyzed with immunoblotting assay using indicated antibodies. **(E)** Cells were treated with various doses of erastin for 24 h. Whole-cell extracts were analyzed using immunoblotting assay with indicated antibodies. **(F)** Cells were treated with erastin alone (10 or 50 μM) for 24 h, TRAIL (1 ng/ml) alone for 4 h, or pretreated with erastin (10 or 50 μM) for 20 h and then exposed to TRAIL (1 ng/ml) for an additional 4 h. Whole-cell lysates were analyzed using immunoblotting assay with indicated antibodies.

Previous studies have shown that inhibition of cystine-glutamate exchange by ferroptotic agents leads to activation of an ER stress response and upregulation of the *CHAC1* (glutathione-specific gamma-glutamylcyclotransferase 1) gene [38, 39]. Data from microarray assay studies (Figure 4A) also reveal that the ferroptotic agent ART promotes the ER stress marker ATF4 (activating transcription factor 4)-dependent gene expression such as TRB3 (*tribbles* homolog 3) and CHOP (C/EBP-homologous protein) [40]. Ferroptotic agent-induced ER stress was confirmed by detecting the unfolded protein response (UPR) by using the anti-ubiquitinylated protein antibody FK2 antibody. Immunoblotting analysis showed that ART- or erastin-treated HCT116 cells contained a higher level of ubiquitin conjugates compared with untreated control cells (Figure 4B). In particular, inhibition of proteasomal degradation flux with MG132 clearly revealed an increase in ubiquitinated proteins in ART- or erastin-treated cells (Figure 4B). These results imply that the UPR occurs during treatment with the ferroptotic agents ART and erastin. Next, we detected ferroptotic agent-induced ER stress by using ER stress markers such as GRP78 (78 kDa glucose-regulated protein) and CHOP. As shown in Figure 4C-4E, a ferroptotic agent-induced ER stress response occurred in a time- and dose-dependent manner. A combined treatment of TRAIL and erastin did not alter the ER stress response (Figure 4F).

### Inhibition of ferroptotic agent-induced lipid peroxidation by ferrostatin-1 and liproxstatin-1 neither blocks the ER stress response nor suppresses the combined treatment of erastin or ART with TRAIL-induced synergistic cytotoxicity

**Figure 5:**
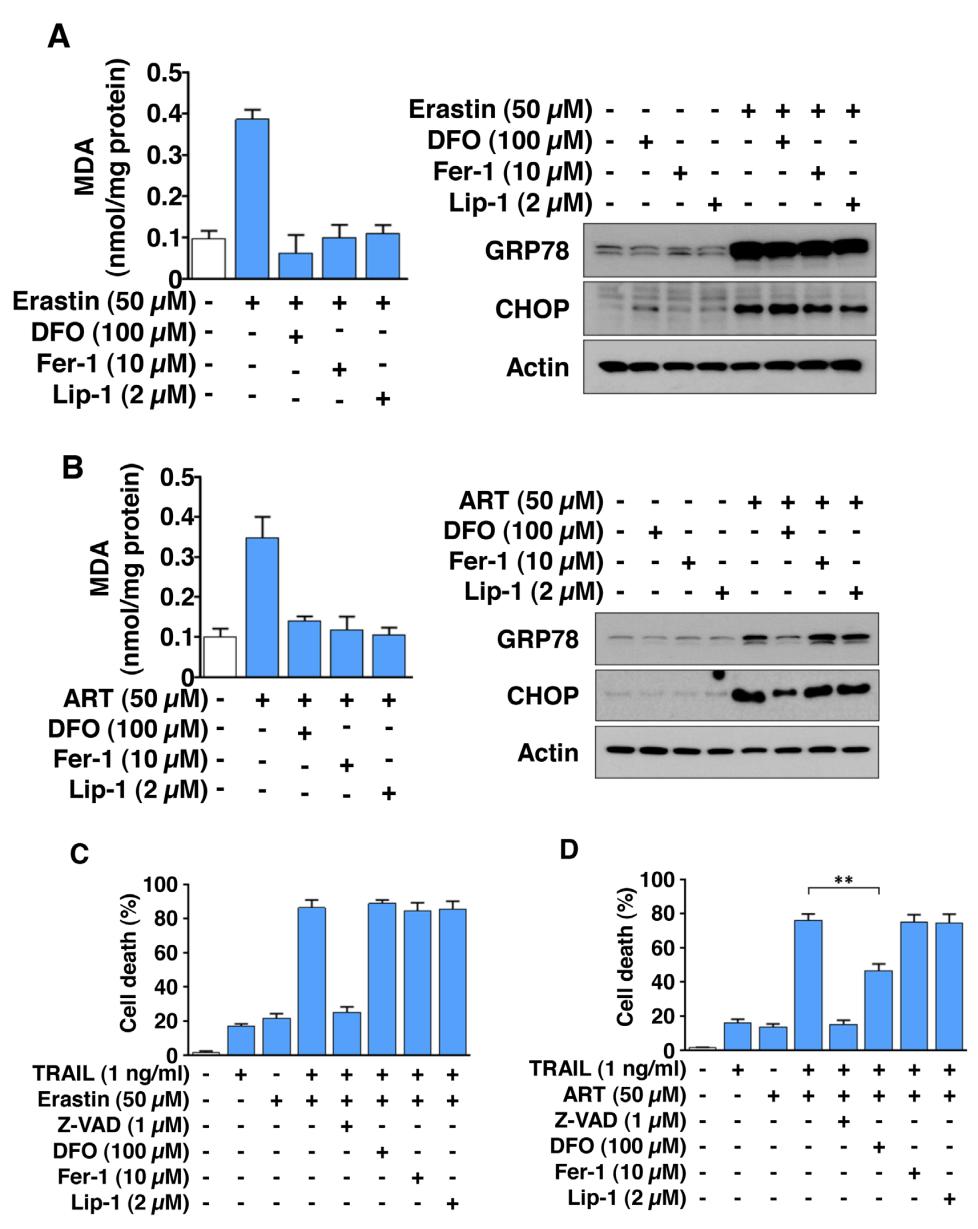
The combinatorial treatment of ferroptotic agent and TRAIL promotes apoptosis via ER stress, but not lipid peroxidation in HCT116 cells **(A** and **B)** Cells were treated with 50 μM erastin (A) or ART (B) for 24 h in the absence/presence of DFO (100 μM), ferrostatin-1 (Fer-1, 10 μM), or liproxstatin-1 (Lip-1, 2 μM). Lipid peroxidation level was detected by MDA assay (left panels) and whole-cell lysates were analyzed with immunoblotting assay using indicated antibodies (right panels). **(C** and **D)** Cells were pretreated with erastin (C) and ART (D) for 20 h in the absence/presence of Z-VAD (1 μM), DFO (100 μM), Fer-1 (10 μM), or Lip-1 (2 μM) and then exposed to TRAIL (1 ng/ml) for an additional 4 h. Cell death was determined using trypan blue exclusion assay and plotted.

We examined whether ferroptotic lipid peroxidation inducing agents play a role in ER stress and the combinatorial treatment-induced synergistic cytotoxicity. To examine this possibility, HCT116 cells were treated with erastin/ART in the absence/presence of the iron chelator deferoxamine (DFO), the lipid peroxidation inhibitors ferrostatin-1 (Fer-1), and liproxstatin-1 (Lip-1). Figure 5A and 5B show that Fer-1 and Lip-1 inhibited erastin/ART-induced lipid peroxidation, but not ER stress. DFO also inhibited erastin-induced lipid peroxidation, but not ER stress. However, unlike erastin, ART-induced ER stress was inhibited by treatment with DFO. DFO, Fer-1, and Lip-1, but not the caspase inhibitor Z-VAD, protected cells from ferroptosis (data not shown) and did not prevent the combinatorial treatment of erastin and TRAIL-induced synergistic cytotoxicity (Figure 5C). Similar results were observed with Fer-1 and Lip-1 on ART- and TRAIL-treated cells (Figure 5D). Unlike erastin and TRAIL-induced synergistic cytotoxicity, ART- and TRAIL-induced synergistic cytotoxicity was inhibited by treatment with DFO. These results indicate that ER stress plays an important role in ferroptotic agent and TRAIL-induced synergistic cytotoxicity.

### Ferroptotic agent induces PUMA expression

**Figure 6:**
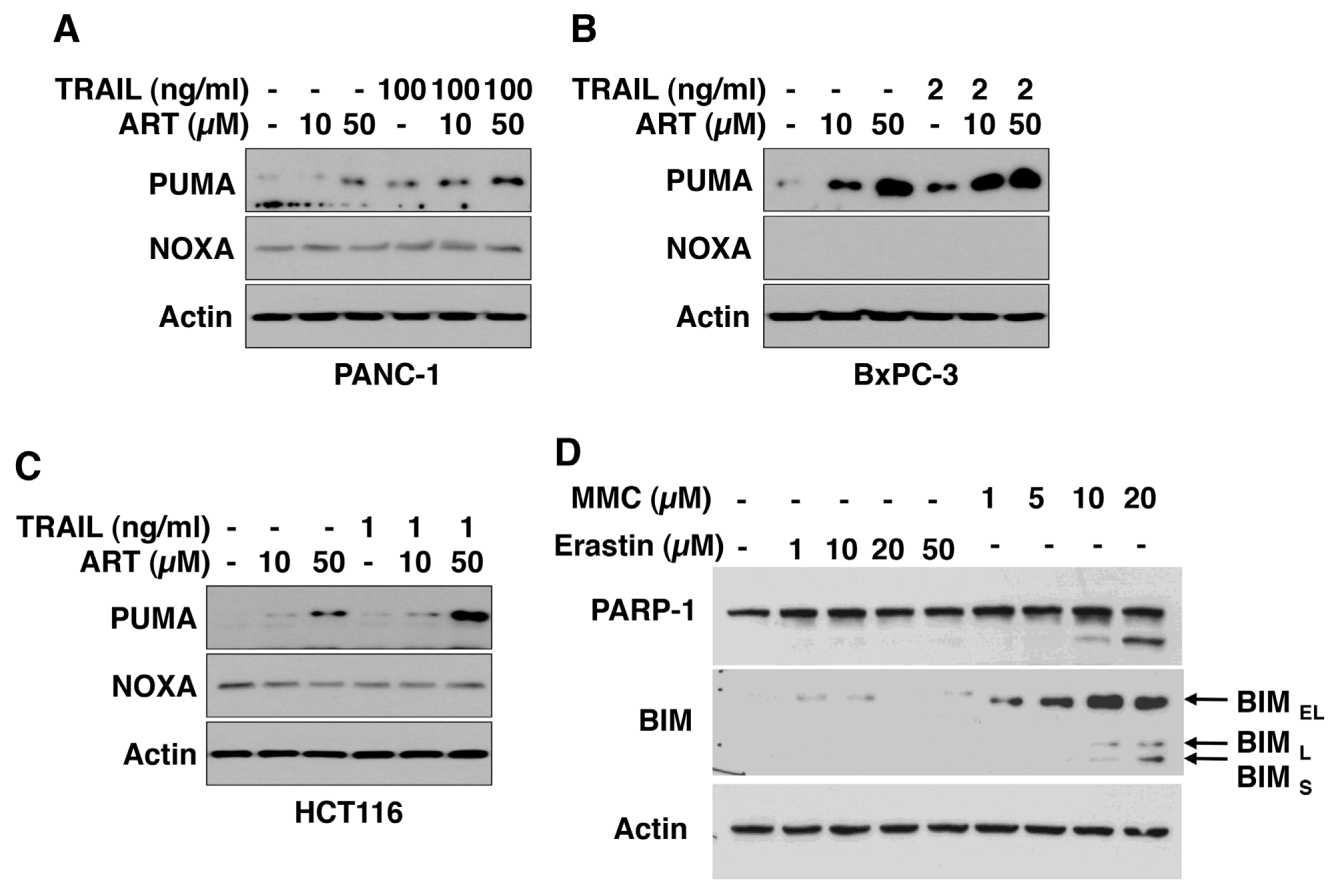
ART increases PUMA protein level PANC-1 **(A)**, BxPC-3 **(B)**, and HCT116 **(C)** cells were pretreated with ART (10 or 50 μM) for 20 h and then exposed to TRAIL (PANC-1, 100 ng/ml; BxPC-3, 2 ng/ml; HCT116, 1 ng/ml) for additional 4 h. Whole-cell lysates were analyzed with immunoblotting assay using indicated antibodies. **(D)** HCT116 cells were treated with erastin (1-50 μM) or mitomycin C (1-20 μM, MMC) for 24 h. Whole-cell lysates were analyzed with immunoblotting assay using indicated antibodies.

Previous studies show that CHOP binds to the pro-apoptotic protein PUMA (p53 upregulated modulator of apoptosis) promoter during ER stress and induces PUMA expression [41]. CHOP also induces several other pro-apoptotic proteins such as NOXA (Latin for *damage*) and BIM [42, 43]. Figure 6 shows that the ferroptotic agent ART induces PUMA expression in three different cancer cell lines. The combinatorial treatment of TRAIL and ART either maintained or enhanced the ER stress response. However, unlike PUMA, we either failed to detect NOXA expression in BxPC-3 cells or did not observe an increase in NOXA expression during ART treatment in HCT116 cells (Figure 6B and 6C). We did not observe an increase in BIM expression during erastin treatment in HCT116 cells. Unlike erastin, the DNA damaging agent mitomycin C induced three isoforms of BIM: BIM_EL_, BIM_L_, and BIM_S_ (Figure 6D).

### Ferroptotic agent promotes TRAIL-induced apoptosis via the p53-independent CHOP/PUMA pathway

**Figure 7:**
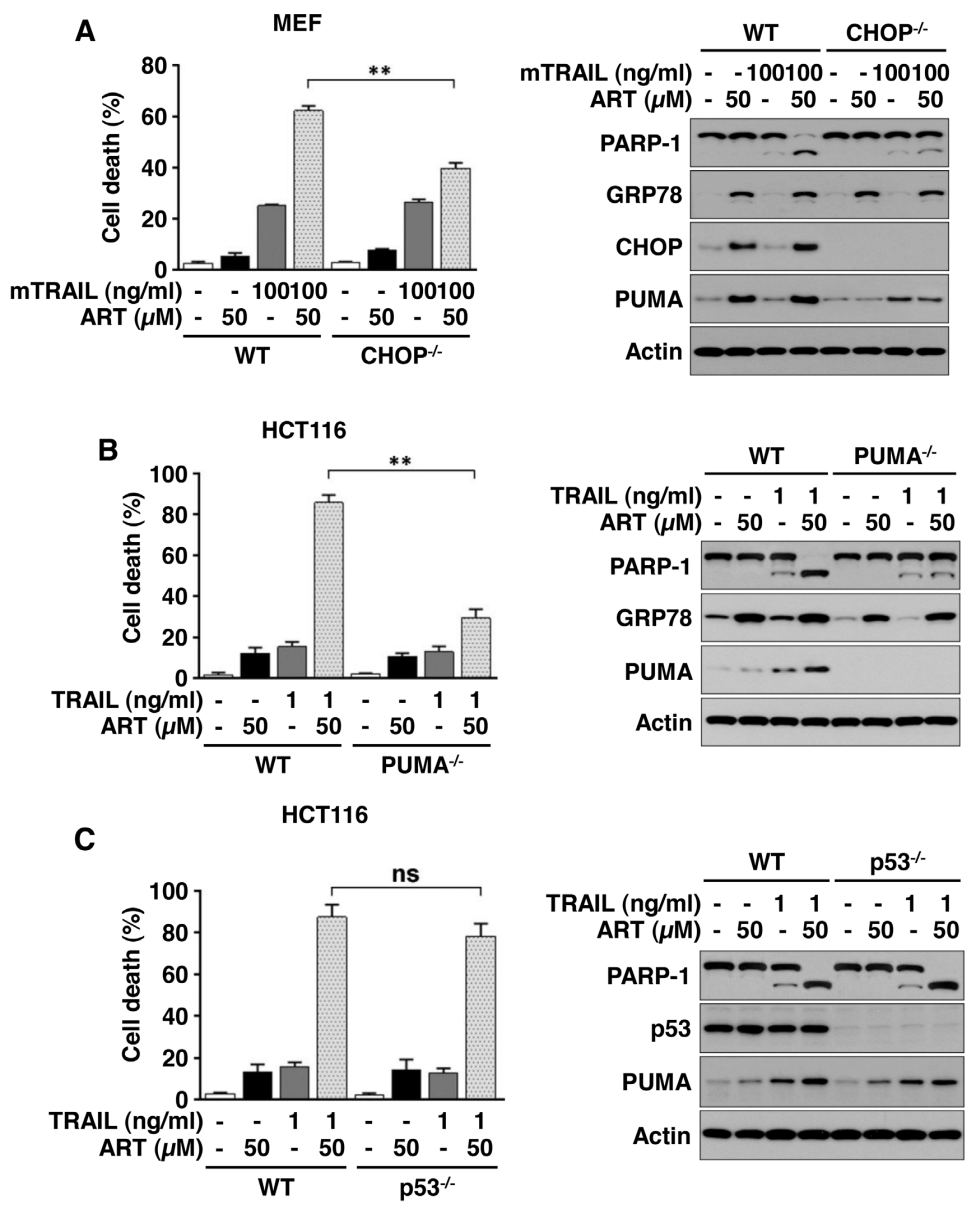
ART promotes TRAIL-induced apoptosis via the p53-independent CHOP/PUMA pathway **(A)** Mouse embryonic fibroblasts (MEFs) wild-type (WT) or MEF CHOP knockout (KO) cells were pretreated with ART (10 or 50 μM) for 20 h and then exposed to recombinant murine TRAIL (mTRAIL; 100 ng/ml) for an additional 4 h. Cell death was determined using trypan blue exclusion assay and plotted (left panel). Whole-cell extracts were analyzed with immunoblotting assay using indicated antibodies (right panel). **(B)** HCT116 WT or HCT116 PUMA KO cells were pretreated with ART (10 or 50 μM) for 20 h and then exposed to TRAIL (1 ng/ml) for additional 4 h. Cell death was determined by counting and plotted (left panel). Whole-cell extracts were then analyzed with immunoblotting using indicated antibodies (right panel). **(C)** HCT116 WT or HCT116 p53 KO cells were pretreated with ART (10 or 50 μM) for 20 h and then exposed to TRAIL (1 ng/ml) for an additional 4 h. Cell death was determined using trypan blue exclusion assay and plotted (left panel). Whole-cell extracts were then analyzed with immunoblotting assay using indicated antibodies (right panel).

We further examined the role of ER stress-associated CHOP and PUMA expression in synergistic apoptosis during the combinatorial treatment of ART and TRAIL. For this study, we employed a CHOP knockout (CHOP^−/−^) mouse embryo fibroblast (MEF) cell line and a PUMA knockout (PUMA^−/−^) HCT116 cell line. As shown in Figure 7A and 7B, combinatorial treatment-induced synergistic apoptosis was suppressed in CHOP^−/−^ MEFs and PUMA^−/−^ cells. Moreover, the combinatorial treatment-induced synergistic apoptosis was not inhibited in p53 knockout (p53^−/−^) cells (Figure 7C). These results suggest that the p53-independent CHOP/PUMA pathway is responsible for the combinatorial treatment-induced synergistic apoptosis.

### Effect of ART and TRAIL on the growth of xenograft tumors

**Figure 8:**
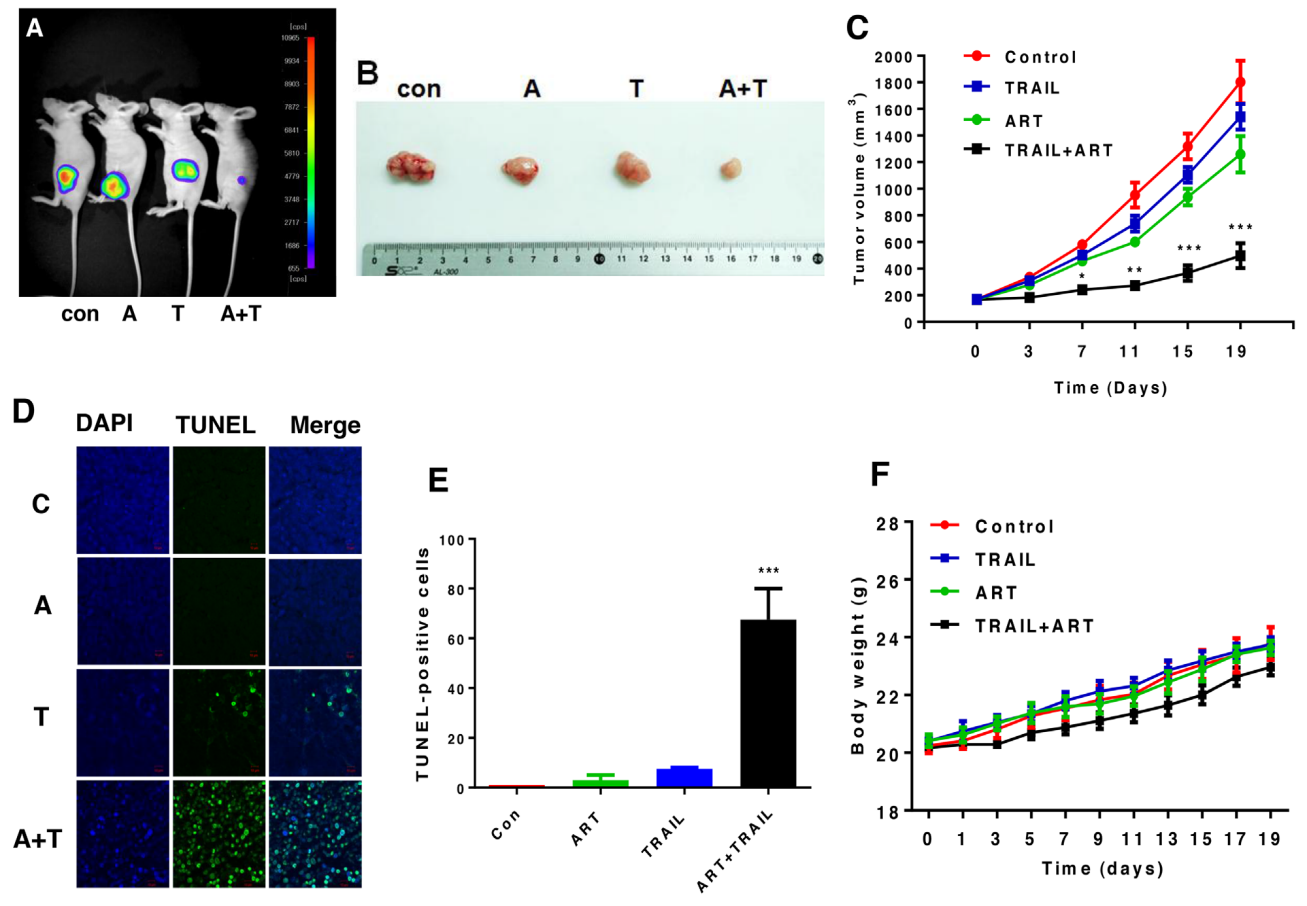
The combinatorial treatment of ART and TRAIL synergistically inhibits tumor growth Nude mice were subcutaneously inoculated with 1×10^6^ HCT116-luc cells. When the tumor volume reached approximately 200 mm^3^, tumor bearing mice were treated with ART (A, 200 mg/kg, twice per week, oral gavage) alone, TRAIL (T, 100 μg/kg, twice per week, intra-tumoral) alone, or the combination of ART and TRAIL (A + T). **(A)** Mice were imaged using the NightOWL LB983 bioluminescence imaging (BLI) system. Representative images are shown on day 19. **(B)** Tumor tissues were harvested on day 19 and displayed. **(C)** Line graph illustrating the tumor volume (mm^3^) in HCT116-luc tumor-bearing mice treated with PBS alone, ART alone, TRAIL alone, or the combination from day 0 to day 19. Error bars represent the mean ± SD from five mice. For statistical analysis, Student’s *t*-test (two-sided, paired) was used. *p*-values: ^*^, 0.05; ^**^, 0.01; ^***^, 0.001. **(D** and **E)** Tumor tissues were harvested on day 19 and subjected to TUNEL assay and DAPI (4′,6-diamidino-2-phenylindole) staining. Cell nuclei were stained with DAPI. Apoptosis was detected using TUNEL assay (D) and percents of TUNEL-positive cells were plotted (E). **(F)** Line graph illustrating the body weight (gram) in HCT116-luc tumor-bearing mice treated with PBS alone, ART alone, TRAIL alone, or the combination from day 0 to day 19.

*In vivo* studies were performed to examine the effect of the combinatorial treatment with ART and TRAIL on the growth of luciferase-expressing HCT116 xenograft tumors. For xenograft tumor formation, five-week-old male nude mice (Balb/c nude) were subcutaneously inoculated with 1 × 10^6^ HCT116-luc cells into the right hind leg. Prior to treatment with ART and TRAIL, tumor size was measured two to three times per week until the volume reached approximately 200 mm^3^. Tumor volume was calculated as W^2^ × L × 0.52, where L is the largest diameter and W is the diameter perpendicular to L. After establishment of these tumor xenografts, mice were randomized into four groups of five mice per group. ART (200 mg/kg, oral gavage administration) and/or rh-TRAIL (100 μg/kg, intratumoral injection) were administered twice per week for two weeks. TRAIL alone did not significantly affect tumor growth compared with the control group. ART alone caused a decrease of tumor growth (p < 0.05). The combinatorial treatment with ART and TRAIL was significantly more effective in inhibiting tumor growth compared with single treatment (p < 0.001) (Figure 8C). Data from terminal deoxynucleotidyl transferase (TdT) dUTP nick-end labeling (TUNEL) assay and quantitative analysis (Figure 8D and 8E) show that the combinatorial treatment effectively induced apoptosis. Although the tendency for weight loss was observed during the combined treatment of ART and TRAIL, it was not statistically significant (Figure 8F).

## DISCUSSION

Several conclusions can be drawn upon consideration of the data presented in the current study. First, the combined treatment of erastin or ART and TRAIL synergistically induced apoptosis, but not ferroptosis. Second, ER stress played an important role in the synergistic apoptosis through activating the p53-independent CHOP-PUMA axis.

It is well known that the ER stress response mediated by the PERK (PKR-like ER kinase)-eIF2α (eukaryotic initiation factor 2α)-ATF4 pathway is involved in regulation of the expression of several target genes such as CHOP [44, 45]. Data from immunoblotting assay confirmed an increase in expression of the ER stress-related genes *GRP78* and *CHOP* during ferroptotic agent treatment (Figures 4, 5, and 7). Several researchers have reported that ER stress induces apoptosis through inducing pro-apoptotic proteins such as PUMA, NOXA, GADD34 (growth arrest and DNA damage-inducible protein), ERO1α (endoplasmic reticulum, oxidoreductin-1α), and BIM (Bcl-2-like protein 11) [41, 42]. However, unlike previous reports, we observed an increase in expression of PUMA, but not NOXA and BIM, during treatment with ferroptotic agent (Figure 6). This discrepancy needs further investigation. Presently, we can only speculate on the differential expression in ER response-inducible proapoptotic gene expression during treatment with ferroptotic agent. Several transcription factors are known to regulate transcription of *PUMA* and *NOXA* gene expression: p53, c-Myc, forkhead box O3A (FoxO3A), p65 or p52 subunit of nuclear factor-κB, p73, CHOP, E2F1, C/EBPβ (CCAAT-enhancer-binding proteins), CREB (cAMP response element-binding protein), c-Jun, and Sp1 for *PUMA* gene expression [46], and HIF-1α, E2F1, c-myc, p53, p73, and ATF4 for *NOXA* gene expression [47]. They may share the same transcription factors for p53-dependent expression of these genes by genotoxic stress, but not in p53-independent expression of these genes by other stimuli.

In the present study, we report that Fer-1 and Lip-1 (inhibitors of ferroptosis), when combined with TRAIL, still suppressed ferroptosis but not synergistic apoptosis and ER stress. However, unlike Fer-1 and Lip-1, the different ferroptosis inhibitor DFO inhibited ART-induced ER stress exclusive of that induced by erastin as well as synergistic apoptosis (Figure 5). This was probably due to differences in mechanisms by which each ferroptosis inducer contributes to the various metabolic derangements that lead ultimately to ER stress. Since DFO has pleiotropic effects (inhibition of iron-mediated lipid peroxidation, inhibition of NF-κB activation, and involvement in several peroxidative systems [48, 49]), DFO may affect ART-induced ER stress and its activation of NF-κB by preventing IκBα degradation [48, 50, 51]. Obviously, further studies are necessary to understand the molecular mechanism of differences between each ferroptosis inducer-induced ER stress.

Although the ferroptotic agent erastin and ART induced pro-apoptotic protein PUMA expression (Figures 6 and 7), unlike in previous reports, erastin and ART did not induce apoptosis; the caspase substrate PARP-1, a hallmark feature of apoptosis, was not cleaved during ferroptotic agent treatment (Figures 1 and 2). These results suggest that ferroptotic agent-induced PUMA remains inactive during treatment with ferroptotic agent alone and then switches to an active state during combinatorial treatment with ferroptotic agent and TRAIL. Previous biochemical studies indicate that PUMA induces apoptosis by activating the multidomain proapoptotic protein Bax and/or Bak through its interaction with antiapoptotic Bcl-2 family members such as Bcl-2 (B-cell lymphoma 2) and Bcl-xL (B-cell lymphoma-extra large), thereby triggering mitochondrial dysfunction, cytochrome *c* release, and caspase activation [46]. How ferroptotic agent-induced PUMA sustains a biochemically inactive state during treatment with ferroptotic agent alone is a question that remains unanswered. It is possible that ferroptotic agent-induced PUMA remains inactive through binding with anti-apoptotic Bcl-2 family members (Bcl-2, Bcl-xL, Mcl-1) as well as Beclin-1. During combinatorial treatment with ferroptotic agent and TRAIL, Beclin-1 is cleaved by TRAIL-activated caspase 8 [52] and the cleavage of Beclin-1 leads to PUMA changing from an inactive to an active state. The conversion of PUMA from an inactive to an active state results in an increase in apoptosis. Another possibility is the apoptotic threshold effect [53]. Apoptosis occurs when an apoptotic threshold is reached, owing to the inhibition of Bcl-2, Bcl-xL, and Mcl-1 by PUMA [53]. A combinatorial treatment of ferroptotic agent and TRAIL induces the level of PUMA at which apoptosis occurs above a threshold. Obviously, these possibilities need to be further examined to understand the role of PUMA in the combinatorial treatment-induced synergistic apoptosis.

## MATERIALS AND METHODS

### Cell lines and cell culture conditions

Human pancreatic cancer PANC-1 and BxPC-3 cells and human colon cancer HCT116 cells were previously obtained from American Type Culture Collection (ATCC, Manassas, VA). PUMA-deficient (PUMA^−/−^) and p53-deificient (p53^−/−^) HCT116 cells were provided by Dr. B. Vogelstein (Johns Hopkins University, Baltimore, MD). CHOP-deficient (CHOP^−/−^) and corresponding wild-type (WT) MEF cell lines were provided by Dr. Randal J. Kaufman (Sanford Burnham Medical Research Institute, CA). Each cell line was maintained in a medium as follows: HCT116 cells in McCoy’s 5A; PANC-1 cells and MEFs in Dulbecco’s Modified Eagle Medium (DMEM); BxPC-3 cells in Roswell Park Memorial Institute (RPMI)-1640 supplemented with 2 mM glutamine. All cell lines were maintained with 10% fetal bovine serum (FBS) and incubated in a humidified atmosphere of 5% CO_2_ at 37°C.

### Chemicals and reagents

For production of recombinant human TRAIL, a human TRAIL cDNA fragment (amino acids 114–281) obtained by RT-PCR was cloned into a pET-23d plasmid (Novagen, Madison, WI), and His-tagged TRAIL protein was purified using the Qiagen express protein purification system (Qiagen, Valencia, CA). Soluble recombinant murine TRAIL and cell-permeant pan caspase inhibitor Z-VAD-FMK were purchased from R&D systems (Minneapolis, MN). Liproxstatin-1, deferoxamine, and ferrostatin-1 were obtained from Sigma-Aldrich (St. Louis, MO). Artesunate, erastin, and mitomycin C were purchased from Selleckchem (Houston, TX).

### Microarray assay

HCT116 cells were treated with 50 μM ART for 24 h and total RNA was extracted using the RNeasy mini kit (Qiagen, Valencia, CA) according to the manufacturer’s instructions. RNA quality and integrity were verified using the Agilent 2100 Bioanalyzer system (Agilent Technologies, Santa Clara, CA). Biotin-labeled cRNA samples for hybridization on Illumina HumanHT-12 v4 Expression BeadChip (Illumina, Inc., San Diego, CA) were prepared according to Illumina’s recommended sample labeling procedure.

### Lipid peroxidation assay

To measure the concentration of MDA, one of the end products of lipid peroxidation, colorimetric analysis of lipid ROS production was carried out using a Lipid Peroxidation (MDA) Assay Kit (#MAK085, Sigma-Aldrich) according to the manufacturer’s instructions. MDA-TBA adduct was quantified colorimetrically at 532 nm using a spectrophotometer.

### Apoptosis assay by flow cytometry

To detect the translocation of phosphatidylserine, one of the markers of apoptosis, from the inner to the outer leaflet of the plasma membrane, cells were stained with Annexin V according to the manufacturer’s protocol of the fluorescein isothiocyanate Annexin V Apoptosis Detection Kit (BD Biosciences, San Diego, CA). Flow cytometry was performed using an Accuri C6 flow cytometer (BD Biosciences).

### Cell death and viability assay

Cell death was typically measured using trypan blue exclusion assay to detect plasma membrane integrity as previously described [54]. For quantification of cell death rate, cells were trypsinized and stained with trypan blue followed by counting with a hemocytometer under microscope. In some experiments, propidium iodide staining was performed. Red-stained cells were considered dead. Quantification of cell death was further confirmed using flow cytometry. Cell viability was typically assessed in 96-well format with Alamar Blue Cell Viability Assay Kit (Invitrogen, Carlsbad, CA) according to the manufacturer’s instructions.

### Western blotting and antibodies

Immunoblotting was carried out as previously described [55]. The following antibodies were used in this study: anti-PARP-1, anti-cleaved caspase-3, anti-cleaved caspase-8, anti-caspase-9, anti-cleaved caspase-9, anti-HO-1, anti-NOXA, anti-CHOP, anti-GRP78, anti-PUMA (Cell signaling Technology, Beverly, MA), anti-actin (MP Biomedicals, Solon, OH), goat anti-rabbit IgG-horseradish peroxidase (HRP), and goat anti-mouse IgG-HRP (Santa Cruz Biotechnology, Santa Cruz, CA).

### Combination index (CI) analysis

CIs were calculated using the CompuSyn software (ComboSyn, Inc., Paramus, NJ, USA). Base on CI values, the extent of synergism/antagonism was determined. In general, CI values below 1 suggest synergy, whereas CI values above 1 indicate antagonism between the drugs. CI values in the 0.9–1.10 range mainly indicate additive effects, those between 0.9–0.85 suggest slight synergy, those in the range of 0.7–0.3 indicate moderate synergy, and those less than 0.3 suggest strong synergy.

### Animal model

Human colon adenocarcinoma HCT116-Luc cells were established by subcutaneously injecting 10^6^ cells into the right hind leg of five-week-old male nude mice (Balb/c nude) (Charles River Labs, Wilmington, MA, USA). Prior to treatment with ART and TRAIL, tumor size was measured two to three times per week until the volume reached approximately 200 mm^3^. Tumor volume was calculated as W^2^ × L × 0.52 where L is the largest diameter and W is the diameter perpendicular to L. After establishment of these tumor xenografts, mice were randomized into four groups of five mice per group. Mice were fed *ad libitum* and maintained in environments with controlled temperature of 22–24°C and 12 h light and dark cycles. Artesunate group: 200 mg/kg –twice per week –oral gavage—two-week treatment rhTRAIL group: 100 ng/g –twice per week—intra-tumoral injection—two-week treatment. Animals were anesthetized and subjected to NightOWL LB983 bioluminescence imaging (BLI) system (Berthold Technologies, TN, USA). D-luciferin sodium salt (BioVision Inc. Milpitas, CA) at 100 mg/kg was administered intraperitoneally as a substrate before BLI imaging. The captured images were quantified using the IndiGo™ software package. All animal procedures were carried out in accordance with guidelines approved by the Korea University Institutional Animal Care and Use Committee (IACUC).

### Statistical analysis

All the values are represented as mean ± S.D. Statistical analysis was performed one-way analysis of variance (ANOVA) followed by Bonferroni’s test or by the Student’s *t*-test as indicated using GraphPad Prism 7 software. *P* value less than 0.05 was defined as statistical significance. Where indicated *p < 0.05; ^**^p < 0.01; ^***^p < 0.001.

## Abbreviations

Apaf1: apoptosis signal-regulating kinase
ART: artesunate
ATCC: American Tissue Type Culture Collection
ATF4: activating transcription factor 4
Bak: Bcl-2 homologous antagonist killer
Bax: Bcl-2–associated X protein
Bcl-2: B-cell lymphoma 2
Bcl-xL: B-cell lymphoma-extra large
BH3: Bcl-2 homology domain 3
Bim: Bcl-2-like protein 11
BLI: bioluminescence imaging
BSO: buthionine sulfoximine
C/EBP: CCAAT-enhancer-binding proteins
CHAC1: glutathione-specific gamma-glutamylcyclotransferase 1
CHOP: CCAAT-enhancer-binding protein homologous protein
CREB: cAMP response element-binding protein
DAPI: 4′,6-diamidino-2-phenylindole
DD: death domain
DFO: deferoxamine
DISC: death-inducing signaling complex
DR: death receptor
DR4: death receptor 4
DR5: death receptor 5
eIF2α: eukaryotic initiation factor 2α
ER: endoplasmic reticulum
ERO1α: endoplasmic reticulum, oxidoreductin-1α
FACS: fluorescence-activated cell sorting
FBS: fetal bovine serum
Fer-1: ferrostatin-1
FITC: fluorescein isothiocyanate
FoxO3A: forkhead box O3A
GADD34: growth arrest and DNA damage-inducible protein
GPX4: glutathione peroxidase 4
GRP78: 78 kDa glucose regulated protein
GSH: glutathione
HO-1: heme oxygenase-1
KI: knock-in
KO: knockout
Lip-1: liproxstatin-1
MDA: malondialdehyde
MEF: mouse embryo fibroblast
PARP: poly (ADP-ribose) polymerase
PBS: phosphate-buffered saline solution
PERK: PKR-like ER kinase
PI: propidium iodide
PUMA: p53 upregulated modulator of apoptosis
RPMI: Roswell Park Memorial Institute medium
ROS: reactive oxygen species
tBid: truncated Bid
TRAIL: tumor necrosis factor-related apoptosis-inducing ligand
TRB3: *tribbles* homolog 3
Ub: ubiquitin
UPR: unfolded protein response
VDAC2/3: voltage-dependent anion channel 2/3
WT: wild-type
