# Correction: Molecular crosstalk between ferroptosis and apoptosis: Emerging role of ER stress-induced p53-independent PUMA expression

**DOI:** 10.18632/oncotarget.25365

**Published:** 2018-05-15

**Authors:** Se Hoon Hong, Dae-Hee Lee, Young-Sun Lee, Min Jee Jo, Yoon A Jeong, William T. Kwon, Haroon A. Choudry, David L. Bartlett, Yong J. Lee

**Affiliations:** ^1^ Department of Surgery, School of Medicine, University of Pittsburgh, Pittsburgh, PA 15213, USA; ^2^ Brain Korea 21 Program for Biomedicine Science, Korea University College of Medicine, Korea University, Seoul 02841, Republic of Korea; ^3^ Division of Oncology/Hematology, Department of Internal Medicine, College of Medicine, Korea University Medical Center, Korea University, Seoul 08308, Republic of Korea

**This article has been corrected:** The correct Figure 2C is given below: The authors declare that this correction does not change the results or conclusions of this paper.

**Figure 2:**
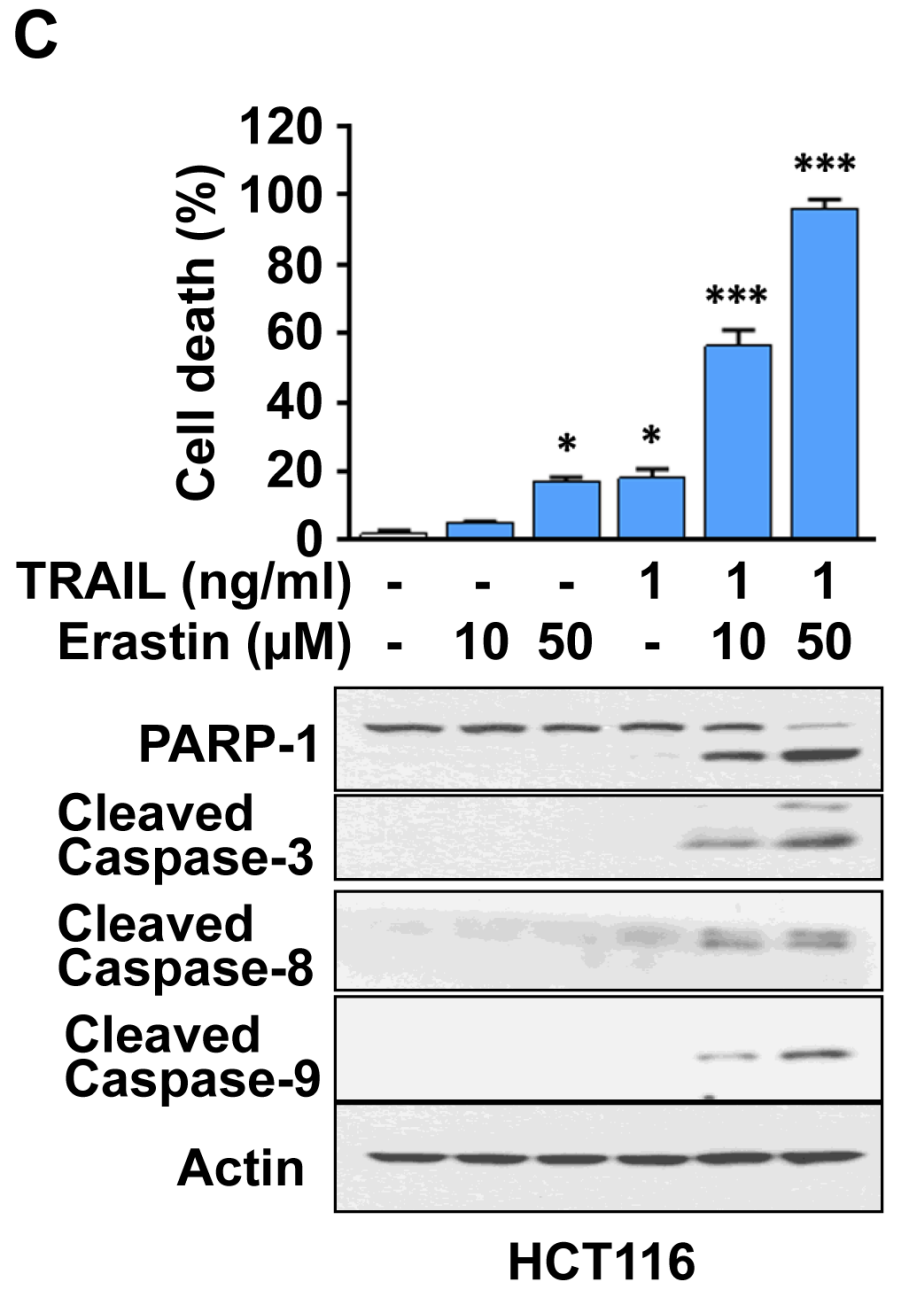
Erastin promotes TRAIL-induced apoptosis **C.** HCT116 cells were pretreated with erastin (10 or 50 µM) for 20 h and then exposed to TRAIL (1 ng/ml) for an additional 4 h. Cell death was determined using trypan blue exclusion assay. Whole-cell extracts were then analyzed with immunoblotting assay using indicated antibodies.

Original article: Oncotarget. 2017; 8:115164-115178. https://doi.org/10.18632/oncotarget.23046

